# The acceptance and willingness to pay (WTP) for hypothetical dengue vaccine in Penang, Malaysia: a contingent valuation study

**DOI:** 10.1186/s12962-018-0163-2

**Published:** 2018-11-22

**Authors:** Hui Yee Yeo, Asrul Akmal Shafie

**Affiliations:** 1 Clinical Research Center, Hospital Seberang Jaya, Jalan Tun Hussein Onn, 13700 Seberang Jaya, Penang Malaysia; 2 Discipline of Social & Administrative Pharmacy, School of Pharmaceutical Sciences, Universiti Sains Malaysia (USM), 11800 Gelugor, Penang Malaysia

**Keywords:** Dengue, Vaccine, Acceptance, Willingness to pay (WTP), Contingent valuation

## Abstract

**Background:**

Malaysia has been experiencing an escalation in dengue cases since the past 5 years. As the dengue vaccine pipeline continues to develop steadily with strong public interests, this study had been sought to elicit the acceptance and the willingness to pay (WTP) for hypothetical dengue vaccine in Malaysia.

**Methods:**

This study adopted the cross-sectional, contingent valuation study that involved 400 respondents in Penang, Malaysia. The double-bounded dichotomous choice via bidding game approach was employed to elicit the WTP value for two hypothetical 3-doses dengue vaccines (Vaccines A and B with 5- and 10-years’ protection, respectively against dengue). A univariate logistic regression model was employed to assess the key determinants of vaccine acceptance, while the mean WTP value and its associated factors were measured by using the parametric two-part model (TPM).

**Results:**

Dengue vaccine appeared to be highly acceptable (88.4%) among the population in Penang, Malaysia. Respondents who were of Chinese ethnicity (OR 0.36, *p *= 0.017), with higher dengue knowledge score (OR 1.43, *p *= 0.016), and higher vaccination attitude score (OR 1.91, *p *< 0.001) were more likely to accept the vaccine. The first step logit estimation from TPM displayed that pensioners (OR 2.37, *p *= 0.036), respondents who were self-employed or working in the private sector (OR 1.21, *p *= 0.002), respondents with higher education level (OR 2.09–3.29, *p *< 0.05), and those who accepted the vaccine (OR 3.23, *p *= 0.001) were more likely to pay for the vaccine. The adjusted mean WTP value for the vaccine was MYR39.21 (USD9.45) per dose. Next, the second-stage regression from TPM revealed the key factors that significantly affected the WTP value, which were composed of age, gender, occupation, household income, dengue prevention practice, and protection duration of the vaccine. The pensioners and those with better dengue prevention practice were willing to pay more for the vaccines. Additionally, all the respondents elicited a higher WTP amount toward the vaccine with longer protection duration (Vaccine B).

**Conclusion:**

Strong acceptance toward dengue vaccine reflects the high value of the vaccine in Malaysia. The WTP estimates offer quantification of the private benefit in reducing occurrences of the disease. Besides, the people’s preferences-based WTP value for the vaccine tends to complement scientific decision-making and prioritization in the management of dengue in the country.

**Electronic supplementary material:**

The online version of this article (10.1186/s12962-018-0163-2) contains supplementary material, which is available to authorized users.

## Background

Dengue disease poses substantial health and economic threats toward those from major tropical and subtropical countries, particularly in Asia, where approximately 265 million cases occurring annually [1]. A staggering 16,729 dengue cases were reported in Malaysia for year 2013, which had increased by four-fold to 73,794 cases in year 2017 [2]. The estimated dengue economic burden in Malaysia appears to fall between USD38.2 million and USD311 million annually [3–5].

At present, the only method implemented to control dengue transmission in Malaysia is through active dengue surveillance and vector control interventions. Malaysia had spent USD73.5 million (0.03% of the country’s GDP) on its National Dengue Vector Control Programme established in year 2010 [6]. The Malaysian Ministry of Health (MOH) regards vector control as a gold standard in its attempt of preventing dengue outbreaks, although vector control has been proven to be partially effective in diminishing the disease burden [7].

The recent introduction of dengue vaccine has offered a new method of preventing dengue transmission. WHO-SAGE published a report [8] that served as the initial position paper on dengue vaccine [9] in year 2016, which recommended to consider the first dengue vaccine (Dengvaxia^®^) in certain geographic settings, where epidemiological data translated high burden of the disease in the age group targeted for vaccination. Dengvaxia^®^ can be found in the market across 14 countries, including four South East Asian countries. Nevertheless, this vaccine has yet to be sold in the Malaysian market as it has been approved to be used solely for Phase IV clinical study [10]. Despite the evidence of its value [11] to the health system, growing interest has been noted in determining its value from the consumers’ perspective. Vaccines that are presently used in MOH Malaysia National Immunization Programme (NIP) have been based on the World Health Organization Expanded Immunization Programme (WHO-EPI). These vaccines are given free-of-charge to all babies at the government healthcare centers, inclusive of the 10 essential vaccine-preventable childhood diseases; Bacillus tuberculosis, diphtheria, tetanus, pertussis, polio, Hepatitis B, mumps, measles, rubella, Japanese Encephalitis, and cervical cancer. Several newer or non-essential vaccines are not covered under the NIP. Thus, the public who would like to be vaccinated with these vaccines would have to pay for the vaccine out-of-pocket.

To date, only five published studies have assessed the aspect of willingness to pay (WTP) for dengue vaccine in Philippines [12], Indonesia [13, 14], Vietnam, Thailand, Colombia [15] and Brazil [16]. However, the Malaysia-specific acceptance toward dengue vaccine and its WTP value have yet to be determined. Therefore, it is crucial at this stage to estimate the acceptance and the WTP of the public prior to officially marketing the vaccine in Malaysia. This serves as a guideline to both the government and the vaccine manufacturers for better planning and decision-making, especially concerning implementation of dengue vaccination in Malaysia. With that, this study looked into the acceptance toward and the WTP for hypothetical dengue vaccines amongst the Malaysian population in Penang state.

## Methods

### Study design, study location and duration

A cross-sectional, contingent valuation (CV) study was performed to estimate the WTP and the factors linked with the WTP amount toward two hypothetical dengue vaccines in Penang, Malaysia. Penang was recorded to be a highly urbanized (90.8%) and the second most densely populated state in Malaysia with a total population of 1.75 million residents in 2015 [17]. The CV refers to a non-market valuation method used to estimate the value of goods placed by individual by using stated preference information, where it measures directly one’s WTP in acquiring a specific good via survey instrument [18]. The respondents were interviewed face-to-face with a validated questionnaire by trained interviewers comprised of final year pharmacy undergraduate students from Universiti Sains Malaysia (USM). The students were given 3-day training prior to data collection. On the first day, they were given an overview of the study objectives, the CV approach, the updates on dengue disease, and the dengue vaccines development. On the second day, they were given detailed explanation for each question and statement embedded in the survey instrument. On the last day, a role-playing exercise was conducted to assess the understanding and the interview techniques of each individual student. The study was conducted over a period of 1 year, from March 2015 until March 2016.

### Population, sample size and sampling procedure

The Krejcie and Morgan sampling method [19] was applied to determine the study sample size. The minimum sample size required was 384 to generate a 95% confidence interval that predicted the characteristics of the population with at least 1 million and a marginal error of ± 5%. Due to the lacking suitable population sampling frame, the respondents were selected using convenient sampling technique. The eligible respondents must be Malaysian aged above 18-years-old who can understand either English or Malay language, and have been residing in Penang for more than 5 years.

### Questionnaire design

The questionnaire used in this study was adapted from the literature [12]. The questionnaire was modified to cover a more comprehensive question variation, to retain interest among respondents and to suit Malaysia’s position. The questionnaire measured the primary outcomes of the study (respondents’ acceptance toward and WTP for dengue vaccine), apart from gathering confounding information pertaining to their demographic background, experience, knowledge and practice towards dengue disease, as well as vaccination attitude. Education level was categorized in accordance to the UNESCO International Standard Classification of Education (ISCED) 2011 [20]. The contents of the questionnaire were validated by an officer from MOH Vector-Borne Disease Division, medical doctors specialized in infectious diseases, local dengue guidelines [21], and academicians from universities. Additionally, two pilot tests were conducted upon the questionnaire to validate its internal consistency of measurements in relation to dengue disease knowledge, household dengue prevention practice, and vaccination attitude by utilizing Cronbach’s alpha reliability test. The questionnaire was designed in English language and translated into Malay language by using the forward–backward and harmonization techniques. The questionnaires are attached in the Additional files 1 and 2.

### Dengue knowledge, household dengue prevention practice and vaccination attitude measures

Dengue knowledge was measured with eight items, where each correct answer was granted a value of 1 point, while 0 for each incorrect answer. The prevention practice was measured with 5-point Likert scale system for five items, with responses of ‘Never’ (1 point), ‘Seldom’ (2 points), ‘Occasionally’ (3 points), ‘Frequently’ (4 points), and ‘All the time’ (5 points). Next, vaccination attitude was measured with 5-point Likert scale system for two items (three items for respondent with children), with responses of ‘Strongly disagree’ (1 point), ‘Disagree’ (2 points), ‘Undecided’ (3 points), ‘Agree’ (4 points), and ‘Strongly agree’ (5 points). Higher scores reflected better dengue knowledge, better household dengue prevention practice, and positive vaccination attitude.

### Willingness to pay measures

Acceptance toward dengue vaccine was measured using a 5-point Likert scale question. The question hypothesized that the vaccine would be completely protective against dengue, 100% safe, and would be provided free by the government. The double-bounded dichotomous-choice and bidding game approach had been applied to elicit WTP and WTP amount from each respondent for two hypothetical dengue vaccines with varied protection durations (5- or 10-year protection for Vaccine A or B, respectively). The initial bidding amount had been based on the outcomes of the recent cost-effectiveness study performed in Malaysia [11]. In order to address the initial bid bias that is commonly linked with CV [22], two sets of questionnaires with varying initial bidding amounts for each vaccine had been prepared (Set 1 with RM40 for Vaccine A, RM80 for Vaccine B, and RM160 for Vaccine B children, while Set 2 with RM80 for Vaccine A, RM120 for Vaccine B, and RM200 for Vaccine B children). Each respondent was randomly assigned to either one of the sets.

**Fig. 1:**
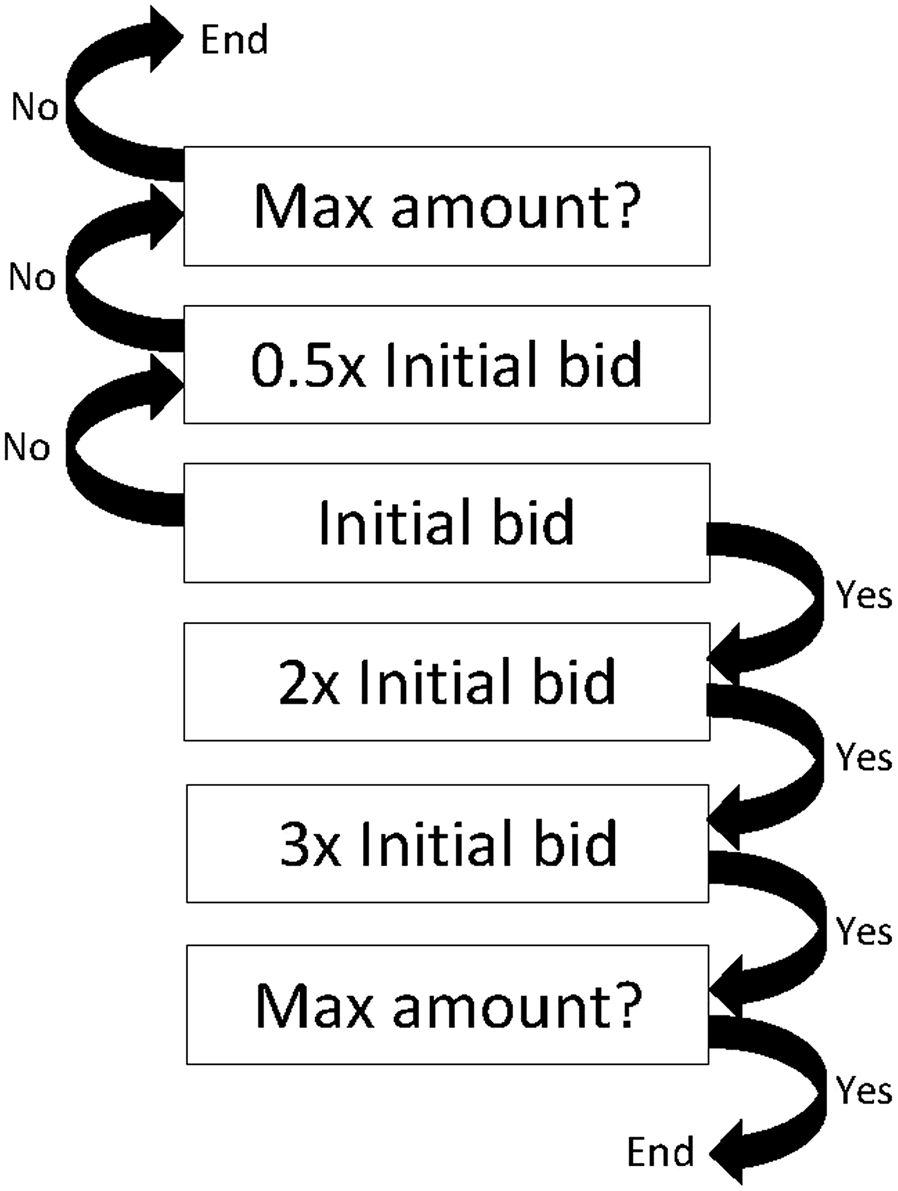
Double-bound dichotomous choice and bidding game approach used for elicitation of WTP amount

Figure 1 illustrates the double-bounded dichotomous choice and the bidding game approach employed in this study. Two to three bids were asked and then followed by an open-ended question that asked the maximum WTP amount for the vaccine. The bidding amount was adjusted accordingly based on the answers provided by the respondents. If the answer was ‘yes’ to the initial bidding amount, the amount was increased one-fold in the subsequent bid. If ‘yes’ was given again, the amount was increased two-fold of the initial bidding amount in the subsequent bid. Nonetheless, if the respondents answered ‘no’ to the initial bidding amount, the subsequent bid was decreased one-fold. The maximum WTP amount was determined as the mid-point between the lower acceptance bid and the higher rejection bid. For those who answered ‘no’ in both bids or ‘yes’ in all bids, an open-ended question that asked for the maximum WTP amount was posed. Those who were not willing to pay any amount at all were directed to indicate their reasons.

### Data analysis

#### Dengue vaccine acceptance

In addition to descriptive statistics, a univariate binary logistic regression model with proportional odds assumption was performed to evaluate the key determinants of acceptance for the vaccine, with the p-value fixed at 0.05, as follows:

$$P\left( Y \right) = \varvec{ }\frac{1}{{1 + \varvec{ }e^{{ - \varvec{ }\left( {b_{0} + \varvec{ }b_{1} x_{1} } \right)}} }}$$

where P(Y) refers to the probability of vaccine acceptance; e denotes the natural logarithm base; b_0_ stands for the interception at y-axis; b_1_ represents the line gradient; and X_1_ predicts the probability of vaccine acceptance.

#### Willingness to pay (WTP) for a hypothetical dengue vaccine

In order to adjust for zero value, a two-part model (TPM) was employed to predict the mean value of WTP amount and its relationship with other covariates. In the TPM, a binary choice model was fitted for the probability of observing a positive-versus-zero outcome in the initial step. Next, in the second step, conditional on a positive outcome, a regression model was fitted for the positive outcome. The model minimized its strategic bias as it permitted the zeros and non-zeros to be generated by varying densities as a special type of mixture model, while the zeros captured were true zeros [23]. Data from the two vaccine scenarios (5- and 10-year protection durations) were combined and run in the TPM. The overall mean value for WTP was calculated as the product of expectations from the first and second parts of the model, as follows:

$${\text{E}}({\text{y}}\left| {{\text{x}}) = \Pr ({\text{y}} > 0\left| {\text{x}} \right.} \right.) \times {\text{E}}({\text{y}}\left| {{\text{y}} > 0,{\text{x}})} \right.$$

where x refers to the vector of explanatory variables; Pr(y > 0|x) is the threshold modelled using logit regression; and E(y|y > 0, x) represent the positives modelled using the ordinary least square (OLS) method.

All the unit costs in this study were valued at 2016 Malaysia Ringgit (MYR) and converted to 2016 US dollar (USD) using the data derived from International Monetary Fund.

## Results

### Socio-demographic profile

A total of 415 respondents were approached, but 15 declined from participating in this study, thus resulting in a 96.4% response rate. The mean age of the respondents was 33 years old (SD 12.0). A majority (52.0%) of the respondents were married, where 45.8% of them claimed to have children (see Table 1). More than half (58.7%) of the respondents had education level 4 and above. Approximately 60% of the respondents reported that they had previous experience with dengue disease.

**Table 1:** Socio-demographic status of the respondents

Characteristics	
Questionnaire, n (%)	
Set A	213 (53.3)
Set B	187 (46.7)
Age (years), mean (SD)	33 (12.0)
Gender, n (%)	
Male	159 (39.7)
Female	241 (60.3)
Ethnicity, n (%)	
Malay	208 (52.0)
Chinese	128 (32.0)
Indian	49 (12.3)
Others	15 (3.7)
Marital status, n (%)	
Single	178 (44.5)
Married	208 (52.0)
Divorced	6 (1.5)
Widow/widower	8 (2.0)
Do you have children?, n (%)	
No	217 (54.3)
Yes	183 (45.7)
Education level (ISCED 2011 level), n (%)	
0	4 (1.0)
1	25 (6.3)
2	83 (20.7)
3	53 (13.3)
> 4	235 (58.7)
Occupation sector, n (%)	
Unemployed	38 (9.5)
Pensioner	16 (4.0)
Student	106 (26.5)
Government	107 (26.7)
Private/Self-employed	133 (33.3)
Monthly household income, n (%)	
≤ MYR 1,000	88 (22.0)
MYR 1,001–2000	62 (15.5)
MYR 2,001–3,000	76 (19.0)
MYR 3,001–4,000	72 (18.0)
MYR 4,001–5,000	40 (10.0)
≥ MYR 5,001	62 (15.5)
Previous experience with dengue, n (%)	
No	158 (39.5)
Yes	242 (60.5)
Duration of interview (minutes), mean (SD)	9.0 (3.0)
Difficulty of the questionnaire, n (%)	
Very difficult	21 (5.3)
Difficult	41 (10.3)
Neutral	136 (34.0)
Easy	151 (37.7)
Very easy	51 (12.7)

### Dengue disease knowledge, household dengue prevention practice, and vaccination attitude

Table 2 summarizes the respondents’ knowledge on dengue disease, household dengue prevention practices, and vaccination attitude. A majority of the respondents displayed a moderate level for knowledge (74%) and household dengue prevention practice (60%) aspects. Meanwhile, a high percentage of the respondents (84%) exhibited good attitude towards vaccination.

**Table 2:** Respondents’ dengue knowledge, household dengue prevention practice and vaccination attitude

Dengue knowledge	Correct answer	Respondent with correct answer n (%)
1. Dengue fever is caused by *Aedes* mosquitoes	Yes	393 (98.3)
2. *Aedes* mosquitos bite during early morning and late evening only	No	92 (23.0)
3. *Aedes* mosquitos breed and lay eggs in stagnant clear water only	No	73 (18.3)
4. One person can be contracted with dengue disease more than once in a lifetime	Yes	241 (60.3)
5. Children are more prone to contracting dengue fever	No	97 (24.3)
6. Dengue fever can be fatal	Yes	373 (93.3)
7. Every person with dengue fever requires blood transfusion	No	136 (34.0)
8. There are specific medicines that can cure dengue disease	No	164 (41.0)

Household dengue prevention practice	Never n (%)	Seldom n (%)	Occasionally n (%)	Frequently n (%)	All the time n (%)
1. We clean and scrub water containers in our house such as vases, flower pot bases and bath tanks	18 (4.5)	62 (15.5)	118 (29.5)	155 (38.7)	47 (11.8)
2. We remove water from items such as unused tyres, empty cans and empty bottles so that they will not become breeding grounds for *Aedes* mosquitoes	21 (5.3)	48 (12.0)	94 (23.4)	169 (42.3)	68 (17.0)
3. I buy insect repellent for my family members to prevent them from contracting dengue	31 (7.8)	30 (7.5)	90 (22.5)	155 (38.7)	94 (23.5)
4. We use mosquito bed nets and window screens in our house	179 (44.7)	67 (16.7)	68 (17.0)	47 (11.8)	39 (9.8)
5. We limit our outdoor activities during early morning and late evening to avoid being bitten by Aedes mosquitos	113 (28.2)	100 (25.0)	88 (22.0)	72 (18.0)	27 (6.8)

Vaccination attitude	Strongly agree n (%)	Disagree n (%)	Undecided n (%)	Agree n (%)	Strongly agree n (%)
1. I think vaccination is important for certain disease prevention	1 (0.3)	10 (2.5)	42 (10.5)	195 (48.7)	152 (38.0)
2. All vaccines registered with Malaysia Ministry of Health (MOH) are safe	3 (0.8)	7 (1.7)	79 (19.7)	189 (47.3)	122 (30.5)
3. I always make sure that my children’s vaccination schedule is met^#^	0 (0.0)	2 (1.1)	21 (13.1)	78 (42.6)	82 (44.8)

^#^Question no 3 was answered by respondents with children only, where n = 183

### Dengue vaccine acceptance

This study discovered that 88.4% of the respondents accepted the vaccine for themselves and 93.6% of the respondents with children accepted to vaccinate their children. The simple logistic regression model (see Table 3) shows that respondents with better vaccination attitude were almost twice more likely to accept dengue vaccine (*p *< 0.01). Respondents with higher dengue knowledge were 1.5 times more likely to accept the vaccine (*p *< 0.05). On the contrary, respondents who were of Chinese ethnicity were less likely to accept the vaccine, in comparison to those of Malay ethnicity (*p *< 0.05). The study outcomes portray that respondents from the Indian and other ethnicities were less likely to accept the vaccine, when compared to those Malay, however, this association was statistically insignificant. Other variables displayed weak correlations with the acceptance of adult dengue vaccine.

**Table 3:** Simple logistic regression results associated with adult dengue vaccine acceptance

Independent variables	Crude Odds Ratio	95% CI OR	*p*-value
Age	1.015	0.96–1.073	0.595
Gender			
Male	–	–	–
Female	0.817	0.386–1.730	0.597
Ethnicity			
Malay	–	–	–
Chinese	0.358	0.154–0.833	0.017*
Indian	0.749	0.227–2.476	0.636
Others	0.403	0.073–2.219	0.296
Marital status			
Single	–	–	–
Married	0.483	0.150–1.561	0.224
Widow/widower	0.317	0.025–3.989	0.374
Have children			
No	–	–	–
Yes	1.530	0.416–5.626	0.522
Education level (ISCED 2011 level)			
0	–	–	–
1	2.992	0.155–57.581	0.468
2	6.700	0.503–89.304	0.150
3	12.256	0.732–205.157	0.081
> 4	6.988	0.512–95.351	0.145
Occupation			
Unemployed	–	–	–
Pensioner	2.227	0.166–29.867	0.546
Student	0.927	0.185–4.643	0.927
Government servant	0.673	0.152–2.985	0.602
Private/Self-employed	1.093	0.268–4.454	0.902
Monthly household income			
≤ RM1000	–	–	–
RM1001–RM2000	0.683	0.170–2.742	0.591
RM2001–RM3000	0.454	0.120–1.726	0.247
RM3001-RM4000	0.495	0.129–1.898	0.305
RM4001-RM5000	1.063	0.177–6.388	0.947
≥ RM5001	0.823	0.173–3.913	0.806
Experience with dengue disease			
No	–	–	–
Yes	1.805	0.846–3.854	0.127
Dengue disease knowledge score	1.426	1.069–1.901	0.016*
Household dengue prevention practice score	1.115	0.994–1.251	0.064
Vaccination attitude score	1.909	1.457–2.501	< 0.001*

* Significant at *p* < 0.05

### Willingness to pay

This study revealed that 36.8%, 26.3%, and 20.8% of the respondents expressed unwillingness to pay for Vaccine A, Vaccine B, and both vaccines, respectively. Most of the respondents who were not willing to pay for the vaccine felt that they would like to have free vaccination from the government or claim from an insurance company (34.5%) or they would rather practice preventive measurements to prevent dengue infection (26.2%). Meanwhile, 20.2% of the respondents stated that they would like to have more information or scientific evidence pertaining to the vaccine, whereas 16.7% of them were not willing to pay for the vaccine because they could not afford the vaccine.

Those who would like to have free vaccination were considered as protest bidders because they placed zero on the vaccine that they had valued. Their zero bids were captured as protest zeros. Respondents who expressed that they could not afford to buy the vaccine or would rather practice preventive measurements had been considered as true zero bidders. This study found equal distribution of protest zero and true zero bids among the respondents who exerted unwillingness to pay for the hypothetical vaccines. By fitting all the observations using the TPM, the adjusted mean score of WTP for the vaccine was MYR39.21 (USD9.45) per dose or MYR117.63 (USD28.36) per vaccinee.

Table 4 summarizes the results from TPM. The logit estimation results showed that respondents with higher education level, pensioners, and private sector employees or self-employed were significantly more likely to pay for the vaccine. From the second-stage regression estimate, respondents who were older, females, or students elicited a lower WTP amount that ranged between MYR2.12 (USD0.51) and MYR54.96 (USD13.25). On the contrary, respondents with higher household prevention practice score and pensioners elicited higher WTP amount of MYR4.96 (USD1.20) and MYR69.09 (USD16.66), respectively. It was observed that the respondents were less likely to pay for Vaccine B, nonetheless, the WTP amount elicited for Vaccine B was RM51.66 higher than the amount for Vaccine A.

**Table 4:** Estimated coefficients of the Two-Parts Model for WTP per dose of dengue vaccine

Independent variable	Willingness to pay?^a^			WTP amount^b^		
	Coefficient	Standard error	*p*-value	Coefficient	Standard error	*p*-value
Age	− 0.0152	0.0141	0.279	− 2.1193	0.9059	0.019*
Gender						
Male	–	–	–	–	–	–
Female	− 0.1589	0.2308	0.491	− 38.1953	12.0638	0.002*
Ethnicity						
Malay	–	–	–	–	–	–
Chinese	0.3222	0.2767	0.244	16.7443	13.1574	0.203
Indian	− 0.3955	0.312	0.205	11.0081	20.0119	0.582
Others	− 0.1273	0.5203	0.807	18.5897	38.4334	0.629
Have children						
No	–	–	–	–	–	–
Yes	0.6508	0.3418	0.057	13.8021	17.0662	0.419
Education level (ISCED 2011 level)						
0	–	–	–	–	–	–
1	2.0927	0.8216	0.011*	83.3829	70.1123	0.234
2	2.8677	0.7849	<0.001*	74.2732	66.4999	0.264
3	3.2913	0. 8174	<0.001*	94.6604	67.7156	0.162
> 4	3.0722	0.7662	<0.001*	113.2698	66.8408	0.090
Occupation						
Unemployed	–	–	–	–	–	–
Pensioner	2.3724	1.1288	0.036*	69.0878	26.0633	0.008*
Student	0.6855	0.4639	0.139	− 54.9633	19.8143	0.006*
Government servant	0.3201	0.3961	0.419	− 19.2953	17.4188	0.268
Private/Self-employed	1.2063	0.3959	0.002*	21.4933	16.0178	0.180
Monthly household income						
≤ RM1000	–	–	–	–	–	–
RM1001–RM2000	− 1.3307	0.399	0.001*	− 51.5993	18.1789	0.005*
RM2001–RM3000	− 0.4423	0.4109	0.282	− 4.9886	15.2938	0.744
RM3001–RM4000	− 1.0823	0.4008	0.007*	4.8979	16.0833	0.761
RM4001–RM5000	− 1.0505	0.4496	0.019*	11.7889	21.4033	0.582
≥ RM5001	0.0101	0.4635	0.983	35.1865	22.8796	0.124
Experience with dengue disease						
No	–	–	–	–	–	–
Yes	0.1046	0.224	0.64	5.1199	11.5706	0.658
Dengue disease knowledge score	− 0.0167	0.8782	0.849	3.1789	4.5639	0.486
Household dengue prevention practice score	0.0634	0.0358	0.076	4.9612	2.0577	0.016*
Vaccination attitude score	0.0364	0.0822	0.658	3.749	3.4303	0.274
Dengue vaccine acceptance						
No	–	–	–	–	–	–
Yes	3.2343	1.002	0.001*	− 38.0342	64.9667	0.558
Vaccine						
A	–	–	–	–	–	–
B	0.6693	0.2309	0.004*	51.655	9.9542	< 0.001*
B for children	1.3994	0.3663	< 0.001*	77.302	15.4756	< 0.001*
Constant	− 5.8708	1.5646	< 0.001*	39.2072	87.9101	0.656

* Significant at P < 0.05; Pseudo R2 = 0.1421

^a^Selection equation using Logit

^b^Regression

## Discussion

Respondents with higher dengue knowledge score and those with better vaccination attitude were more likely to accept the vaccine. This makes sense because most of them perceived that vaccination is essential for disease prevention and thus, were more able to weigh the benefits of dengue vaccination against the risk of dengue infection. Similar association was also reported in Bandung, Indonesia [13]. This study discovered that the respondents’ age, gender, education level, occupation, household income, dengue prevention practice, and dengue vaccine acceptance significantly influenced their WTP and the WTP amount for the vaccine. It is worth highlighting that the WTP amount reflected the value people placed on avoiding risks related to dengue fever. Specific groups of people, particularly those who felt more vulnerable and perceived a higher need to protect themselves (pensioners and those with higher education level), placed higher value for the vaccine to avoid being infection.

Past studies [12–16] investigated the aspect of WTP by using the CV approach. Similar to the study carried out in Bandung, Indonesia [13], this study evaluated WTP by using parametric model. Meanwhile, the other studies probed into WTP values by applying both parametric and non-parametric models. The advantages of non-parametric model lie in their simplicity and transparency. Nonetheless, these estimates offer a relatively lower bound on WTP. On the other hand, the non-parametric model only provides estimates of a fraction of distribution that falls into pre-defined intervals and unsuitable for covariate analysis. The parametric approach allows the assessment on the factors that affect WTP values [24]. The WTP for dengue vaccine reported in this study (USD 28.36 per vaccinee) is comparable to that recorded in Philippines (USD27–USD32 per vaccinee) [12]. This could perhaps due to the similarity shared by Malaysia and Philippines for their annual GDP. However, the adjusted mean score for WTP value in this study was lower when compared to Vietnam (USD24.46), Thailand (USD47.26), and Columbia (USD30.45) [15], but higher in comparison to Indonesia (USD1.94–USD4) [13, 14]. Nevertheless, the study findings cannot be generalized across countries as each study presented different hypothetical vaccine scenarios in the CV and each study evaluated the WTP using different parametric or non-parametric models.

The main limitation in this study resides in the potential biases that often accompany the stated preference models. Strategic bias, such as protest bids or free-rider problem, can overestimate or underestimate the true value [22]. In the WTP analysis, TPM was selected over other models because TPM does not make any assumption on the correlation between the errors of binary and continuous equations. The Monte Carlo evidence shows that when data are generated from a generalized logit model without excluding restrictions to identify the “zeros” equation, the TPM generally produces better estimates of the conditional mean and of marginal effects than the correctly specified generalized logit model [23]. As this study applied the bidding game approach, initial bid bias could have occurred when the respondents’ valuation was affected by the starting WTP amount. Nevertheless, the presence of two different starting WTP amounts for each hypothetical vaccine in this study had greatly reduced such bias effect.

Second, due to financial and time constrains, this study was conducted only in Penang, one of thirteen states in Malaysia, which might not be the actual representation of the diversity in Malaysia. Nonetheless, Penang, being the second most densely populated state in Malaysia with high level of urbanization [25] was believed to be able to represent most, if not all, states in Malaysia where risk of dengue is highly endemic. Additionally, the socio-demographic status of the respondents in this study closely resembled the socio-demographic status of Malaysia, where Malay is the major ethnic constituent, followed by Chinese and Indian.

Finally, due to lack of a population-level sampling frame, the respondents in this study were selected via convenience sampling. This could have led to under-representation or over-representation of certain groups in the study. More rigorous population sampling method, such as stratified cluster sampling using data from Malaysia’s Department of Statics census, should be employed in future research.

## Conclusion

This study has successfully determined the acceptance and WTP value for dengue vaccine amidst the general population in Penang state, Malaysia. The study outcomes signified that the hypothetical dengue vaccine was highly accepted by the respondents (88.4%). The adjusted mean score of the WTP elicited for the hypothetical dengue vaccines was MYR39.21 (USD9.45) per dose or MYR117.63 (USD28.36) per vaccinee.

The WTP estimates offered quantification of the private benefit in reducing the risk of the dengue. For countries where dengue vaccine is only available in the private market, more efforts are warranted to improve knowledge and awareness of dengue risks among the public. Although there is room for the private market for dengue vaccine, the herd protection offered by it should merit it as a public good with exceptional value in public program.

Vaccination against dengue disease could complement the existing surveillance and vector control in curbing economic and health burden of the disease. The strong acceptance toward dengue vaccine among Malaysians portrays the high value of the vaccine in Malaysia. The study outcomes suggest the possibility that a private market for dengue vaccines does exist in Malaysia. Nevertheless, a more robust WTP analysis should be explored in a larger scale using this study as the basis. Although the findings presented in this study deserve further investigation, the people’s preference-based WTP value for the vaccine elicited from CV complements scientific decision-making and prioritization in the healthcare sectors for the country in the future.

## Additional files

**Additional file 1.** Questionnaire Set A.

**Additional file 2.** Questionnaire Set B.

## Abbreviations

CV: contingent valuation
DC: dichotomous choice
GDP: Gross Domestic Products
MOH: Ministry of Health
TPM: two-part model
WTP: willingness to pay
