# Supplementary material for: The acceptance and willingness to pay (WTP) for hypothetical dengue vaccine in Penang, Malaysia: a contingent valuation study
Source: Cost Eff Resour Alloc. 2018 Nov 22;16:60. doi: 10.1186/s12962-018-0163-2 (PMC6249975; doi:10.1186/s12962-018-0163-2)
Supplement: Supplementary file 1 — Additional file 1. Questionnaire Set A. [file 12962_2018_163_MOESM1_ESM.pdf]

No: Set A

Start time:

End time:

<u>Section 1: Demographic</u>

<u>Bahagian 1: Demografik</u>

In order for us to understand your response better, we would like to know more about you. Please fill or check (√) one ONLY at the appropriate option below:
Untuk memahami respon anda dengan lebih baik, kami ingin mengenali anda dengan lebih mendalam. Sila isikan maklumat anda dan letakkan (√) hanya sekali pada ruangan yang disediakan.

1. Age/ *Umur*: __________ years/ *tahun*

2. Gender/ *Jantina*:
   Male/ *Lelaki* [ ]   Female/ *Perempuan* [ ]

3. Ethnicity/ *Kaum*:
   Malay/ *Melayu* [ ]   Chinese/ *Cina* [ ]
   Indian/ *India* [ ]   Others/ *Lain-lain* [ ]

4. Marital status/ *Status perkahwinan*:
   Single/ *Bujang* [ ]   Divorced/ *Bercerai* [ ]
   Married/ *Berkahwin* [ ]   Widow/Widower/ *Janda/ Duda* [ ]

5. Do you have any children?
   Adakah anda mempunyai anak?
   Yes/ *Ya* [ ]   No/ *Tidak* [ ]
   ⬇
   If yes, how many/ *Jika ya, berapakah*:
   Above 13 years old/ *Umur 13 tahun ke atas*: _______
   Below 13 years old/ *Umur 13 tahun ke bawah*: _______

6. Highest qualification/ *Pencapaian akademik tertinggi*:
   No formal education/ *Tiada pendidikan formal* [ ]
   Primary level/ *Tahap primer* [ ]
   Secondary level/ *Tahap sekunder* [ ]
   STPM/ Matriculation/Pre-University/A Level [ ]
   STPM/ Matrikulasi/Pra-Universiti/A Level
   College or University/ *Kolej atau university* [ ]

7. Occupation/ *Pekerjaan*:
   Unemployed/ *Tidak bekerja* [ ]   Pensioner/ *Pesara* [ ]
   Student/ *Pelajar* [ ]   Government Servant/ *Bekerja dengan kerajaan* [ ]
   Private or Self-employed/ *Sektor awam atau Bekerja sendiri* [ ]

8. What is your monthly household income?
   Berapakah pendapatan bulanan rumah tangga anda?
   ≤ RM500 [ ]   RM3001-RM3500 [ ]
   RM501-RM1000 [ ]   RM3501-RM4000 [ ]
   RM1001-RM1500 [ ]   RM4001-RM4500 [ ]
   RM1501-RM2000 [ ]   RM4500-RM5000 [ ]
   RM2001-RM2500 [ ]   ≥ RM5000 [ ]
   RM2501-RM3000 [ ]

9. Have you suffered from dengue disease before?
Pernahkah anda menghidapi penyakit denggi?
Yes/*Ya* [ ] No/*Tidak* [ ]

10. Do you know anyone (family members, relatives and friends) who has suffered from dengue disease before?
Adakah anda mengenali sesiapa (ahli keluarga, saudara-mara dan rakan) yang pernah menghidapi penyakit denggi?
Yes/*Ya* [ ] No/*Tidak* [ ]

11. Do you know anyone (family members, relatives and friends) who has died from dengue disease before?
Adakah anda mengenali sesiapa (ahli keluarga, saudara-mara dan rakan) yang pernah meninggal dunia disebabkan penyakit denggi?
Yes/*Ya* [ ] No/*Tidak* [ ]

Section 2a: Dengue Disease Knowledge

Bahagian 2a: Pengetahuan tentang Penyakit Denggi

1. Dengue fever is caused by *Aedes* mosquitoes.
   Demam denggi adalah penyakit yang dijangkiti oleh nyamuk Aedes.

   ☐Yes/ *Ya* ☐No/ *Tidak* ☐ I don't know/ *Tidak tahu*

2. Aedes mosquitos bite during early morning and late evening only.
   Nyamuk Aedes gigit mangsanya pada waktu awal pagi dan lewat petang sahaja.

   ☐Yes/ *Ya* ☐No/ *Tidak* ☐ I don't know/ *Tidak tahu*

3. Aedes mosquitos breed and lay eggs in stagnant clear water only.
   Nyamuk Aedes membiak dan bertelur di dalam air takungan jernih sahaja.

   ☐Yes/ *Ya* ☐No/ *Tidak* ☐ I don't know/ *Tidak tahu*

4. One person can be contracted with dengue disease more than once in a lifetime.
   Seseorang boleh mendapat penyakit denggi lebih daripada satu kali dalam seumur hidupnya.

   ☐Yes/ *Ya* ☐No/ *Tidak* ☐ I don't know/ *Tidak tahu*

5. Children are more prone to contracting dengue fever.
   Kanak-kanak adalah lebih mudah dijangkiti demam denggi.

   ☐Yes/ *Ya* ☐No/ *Tidak* ☐ I don't know/ *Tidak tahu*

6. Dengue fever can be fatal.
   Demam denggi boleh membawa maut.

   ☐Yes/ *Ya* ☐No/ *Tidak* ☐ I don't know/ *Tidak tahu*

7. Every person with dengue fever requires blood transfusion.
   Setiap orang yang menghidapi demam denggi memerlukan transfusi darah.

   ☐Yes/ *Ya* ☐No/ *Tidak* ☐ I don't know/ *Tidak tahu*

8. There are specific medicines that can cure dengue disease.
   Terdapat ubat-ubat tertentu yang dapat merawat penyakit denggi.

   ☐Yes/*Ya* ☐No/*Tidak* ☐ I don't know/*Tidak tahu*

Section 2b: Household Dengue Prevention Practice
Bahagian 2b : Amalan Pencegahan Denggi Di Rumah

1. We clean and scrub water containers in our house such as vases, flower pot bases and bath tanks.
   Kami membersih dan memberus tempat-tempat pertakungan air di rumah seperti pasu bunga, alas pasu bunga dan kolah mandi.

Never / *Tidak pernah*	Seldom / *Jarang*	Occasionally / *Kadang-kadang*	Frequently / *Kerap*	All the time / *Setiap masa*
1	2	3	4	5

2. We remove water from items such as unused tyres, empty cans and empty bottles so that they will not become breeding grounds for *Aedes* mosquitoes.
   Kami pastikan tempat bertakung air seperti tayar terpakai, tin kosong dan botol kosong dibuang untuk mengelakkan dijadi tempat pembiakan Aedes.

Never / *Tidak pernah*	Seldom / *Jarang*	Occasionally / *Kadang-kadang*	Frequently / *Kerap*	All the time / *Setiap masa*
1	2	3	4	5

3. I buy insect repellent for my family members to prevent them from contracting dengue.
   Saya membeli penyembur serangga untuk ahli-ahli keluarga saya bagi mencegah mereka daripada dijangkiti demam denggi.

Never / *Tidak pernah*	Seldom / *Jarang*	Occasionally / *Kadang-kadang*	Frequently / *Kerap*	All the time / *Setiap masa*
1	2	3	4	5

4. We use mosquito bed nets and window screens in our house.
   Kami memasang kelambu dan jaring tingkap penghalang nyamuk di rumah.

Never / *Tidak pernah*	Seldom / *Jarang*	Occasionally / *Kadang-kadang*	Frequently / *Kerap*	All the time / *Setiap masa*
1	2	3	4	5

5. We limit our outdoor activities during early morning and late evening to avoid being bitten by *Aedes* mosquitos.
   Kami menghadkan aktiviti-aktiviti di luar rumah pada waktu awal pagi dan lewat petang bagi mengelakkan daripada digigit nyamuk Aedes.

Never / *Tidak pernah*	Seldom / *Jarang*	Occasionally / *Kadang-kadang*	Frequently / *Kerap*	All the time / *Setiap masa*
1	2	3	4	5

Section 3a: Vaccination Attitude
Bahagian 3a: Sikap terhadap Vaksinasi

1. I think vaccination is important for certain disease prevention.
   Saya rasa vaksinasi adalah penting untuk mencegah sesetengah penyakit.

Strongly disagree<br>*Sangat tidak setuju*	Disagree<br>*Tidak setuju*	Undecided<br>*Tidak pasti*	Agree<br>*Setuju*	Strongly agree<br>*Sangat setuju*
1	2	3	4	5

2. All vaccines registered with Malaysia Ministry of Health (MOH) are safe.
   Semua vaksin didaftar dengan Kementerian Kesihatan Malaysia (KKM) adalah selamat.

Strongly disagree<br>*Sangat tidak setuju*	Disagree<br>*Tidak setuju*	Undecided<br>*Tidak pasti*	Agree<br>*Setuju*	Strongly agree<br>*Sangat setuju*
1	2	3	4	5

** Questions 3 for those who has children/ *Soalan 3 untuk mereka yang mempunyai anak.***

3. I always make sure that my children's vaccination schedule is met.
   Saya memastikan anak saya mengambil vaksin mengikuti jadual vaksinasi yang ditetapkan.

Strongly disagree<br>*Sangat tidak setuju*	Disagree<br>*Tidak setuju*	Undecided<br>*Tidak pasti*	Agree<br>*Setuju*	Strongly agree<br>*Sangat setuju*
1	2	3	4	5

Section 3b: Dengue Vaccine Acceptance
Bahagian 3b: Tahap PenerimaanVaksin Denggi

1. If a 100% safe and fully protective dengue vaccine is provided free by the government, how likely would you vaccinate yourself?
   Jika kerajaan menawarkan vaksin denggi yang 100% selamat dan berkesan secara percuma, adakah anda akan memvaksinkan diri anda?

Very unlikely<br>*Sangat tidak mungkin*	Somewhat unlikely<br>*Agak tidak mungkin*	Undecided<br>*Tidak pasti*	Somewhat likely<br>*Agak mungkin*	Very likely<br>*Sangat mungkin*
1	2	3	4	5

** Questions 2 for those who has children/ *Soalan 2 untuk mereka yang mempunyai anak.***

2. If a 100% safe and fully protective dengue vaccine is provided free by the government, how likely would you vaccinate your children?
   Jika kerajaan menawarkan vaksin denggi yang 100% selamat dan berkesan secara percuma, adakah anda akan memvaksinkan anak anda?

Very unlikely<br>*Sangat tidak mungkin*	Somewhat unlikely<br>*Agak tidak mungkin*	Undecided<br>*Tidak pasti*	Somewhat likely<br>*Agak mungkin*	Very likely<br>*Sangat mungkin*
1	2	3	4	5

<u>Section 3c: Dengue Vaccine Willingness To Pay (WTP)</u>
<u>Bahagian 3c: Kesanggupan Membayar untuk Vaksin Denggi</u>

Now, please assume that **TWO** different vaccines have been successfully marketed against dengue disease. All the vaccines has to be **PAID FULLY FROM YOUR POCKET**;
Sekarang, sila andaikan terdapatnya ***DUA*** *vaksin yang telah berjaya dipasarkan untuk mencegah penyakit denggi. Semua vaksin tersebut perlu* ***DIBAYAR DI BAWAH TANGGUNGAN SENDIRI****.*

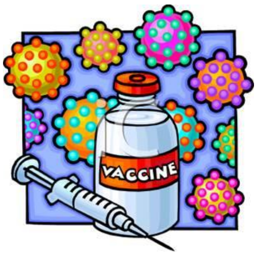	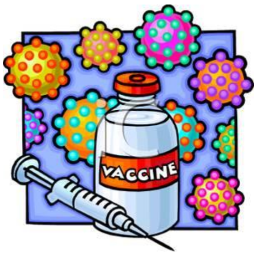
**<u>Vaccine A</u>**	**<u>Vaccine B</u>**
✓ 3-doses vaccine<br>*Vaksin 3-dos*<br>✓ **<u>5 years</u>** protection against dengue disease<br>*Perlindungan terhadap penyakit denggi selama* ***<u>5 tahun</u>***<br>✓ Slight fever and pain at injection site<br>*Demam ringan dan sakit di bahagian suntikan*	✓ 3-doses vaccine<br>*Vaksin 3-dos*<br>✓ **<u>10 years</u>** protection against dengue disease<br>*Perlindungan terhadap penyakit denggi selama* ***<u>10 tahun</u>***<br>✓ Slight fever and pain at injection site<br>*Demam ringan dan sakit di bahagian suntikan*

Which vaccine would you buy?
Vaksin manakah yang anda akan beli?

☐ Vaccine A/ *Vaksin A*

☐ Vaccine B/ *Vaksin B*

☐ Not willing to buy/ *Enggan membeli*
Please proceed to No. 14
Sila teruskan ke No.14

If only dengue vaccine A is available,
Sekiranya hanya Vaksin Denggi A yang ada,

1. Would you purchase dengue vaccine A for yourself?
Adakah anda sanggup membeli vaksin A untuk diri anda sendiri?

☐Yes/*Ya*
If yes, please proceed to No.2
Jika ya, sila teruskan ke No. 2

☐No/*Tidak*
If no, please proceed to No. 7
Jika tidak, sila teruskan ke No.7

2. Would you be willing to pay RM40 per dose for dengue vaccine A?
Adakah anda sanggup membayar RM40 setiap dos untuk vaksin denggi A?

☐Yes/*Ya*
If yes, please proceed to No.3
Jika ya, sila teruskan ke No. 3

☐No/*Tidak*
If no, please proceed to No.5
Jika tidak, sila teruskan ke No.5

3. Would you be willing to pay RM80 per dose for dengue vaccine A?
Adakah anda sanggup membayar RM80 setiap dos untuk vaksin denggi A?

☐ Yes/*Ya*
If yes, please proceed to No.4
Jika ya, sila teruskan ke No.4

☐ No/*Tidak*
If no, please proceed to No.8
Jika tidak, sila teruskan ke No.8

4. Would you be willing to pay RM120 per dose for dengue vaccine A?
Adakah anda sanggup membayar RM120 setiap dos untuk vaksin denggi A?

☐ Yes/*Ya*
If yes, please proceed to No.6
Jika ya, sila teruskan ke No.6

☐ No/*Tidak*
If no, please proceed to No.8
Jika tidak, sila teruskan ke No.8

5. Would you be willing to pay RM 20 per dose for dengue vaccine A?
Adakah anda sanggup membayar RM20 setiap dos untuk vaksin denggi A?

☐ Yes/*Ya*
If yes, please proceed to No.8
Jika ya, sila teruskan ke No.8

☐ No/*Tidak*
If no, please proceed toNo.6
Jika tidak, sila teruskan ke No.6

6. What is the maximum amount you are willing to pay for the vaccine per dose?
Apakah harga maksimum yang anda sanggup bayar untuk setiap dos vaksin denggi tersebut?

____

7. Please state the reason why you refuse to buy the vaccine.
Sila nyatakan sebab anda enggan membeli vaksin denggi tersebut.

☐ I would like to have free vaccination from government or claim from insurance company.
Saya ingin dapatkan vaksin percuma daripada kerajaan atau tuntut daripada syarikat insurans.

☐ I would like to have more information or scientific evidence on the vaccine.
Saya ingin dapatkan maklumat lanjut atau bukti saintifik tentang vaksin ini.

☐ I cannot afford to buy the vaccine. / *Saya tidak mampu untuk membeli vaksin ini*.

☐ I would rather practice preventive measurements to prevent dengue infection.
Saya lebih sanggup mengamalkan langkah-langkah pencegahan daripada dijangkiti penyakit denggi.

☐ Others, please specify: / *Lain-lain, sila nyatakan:*

____

If only dengue vaccine B is available,
Sekiranya hanya Vaksin Denggi B yang ada,

8. Would you purchase dengue vaccine B for yourself?
Adakah anda sanggup membeli vaksin B untuk diri anda sendiri?

☐ Yes/*Ya*
If yes, please proceed to No.9
Jika ya, sila teruskan ke No.9

☐ No/*Tidak*
If no, please proceed to No.14
Jika tidak, sila teruskan ke No.14

9. Would you be willing to pay RM80 per dose for dengue vaccine B?
Adakah anda sanggup membayar RM80 setiap dos untuk vaksin denggi B?

☐ Yes/*Ya*
If yes, please proceed to No.10
Jika ya, sila teruskan ke No.10

☐ No/*Tidak*
If no, please proceed to No.12
Jika tidak, sila teruskan ke No.12

10. Would you be willing to pay RM160 per dose for dengue vaccine B?
Adakah anda sanggup membayar RM160 setiap dos untuk vaksin denggi B?

☐ Yes/*Ya*
If yes, please proceed to No.11
Jika ya, sila teruskan ke No.11

☐ No/*Tidak*
If no, please proceed to No.15
Jika tidak, sila teruskan ke No.15

11. Would you be willing to pay RM240 per dose for dengue vaccine B?
Adakah anda sanggup membayar RM240 setiap dos untuk vaksin denggi B?

☐ Yes/*Ya*
If yes, please proceed to No.13
Jika ya, sila teruskan ke No.13

☐ No/*Tidak*
If no, please proceed to No.15
Jika tidak, sila teruskan ke No.15

12. Would you be willing to pay RM40 per dose for dengue vaccine B?
Adakah anda sanggup membayar RM40 setiap dos untuk vaksin denggi B?

☐ Yes/*Ya*
If yes, please proceed to No.15
Jika ya, sila teruskan ke No.15

☐ No/*Tidak*
If no, please proceed toNo.13
Jika tidak, sila teruskan ke No.13

13. What is the maximum amount you are willing to pay for the vaccine per dose?
Apakah harga maksimum yang anda sanggup bayar untuk setiap dos vaksin denggi tersebut?

____

14. Please state the reason why you refuse to buy the vaccine.
Sila nyatakan sebab anda enggan membeli vaksin denggi tersebut.

☐ I would like to have free vaccination from government or claim from insurance company.
Saya ingin dapatkan vaksin percuma daripada kerajaan atau tuntut daripada syarikat insurans.

☐ I would like to have more information or scientific evidence on the vaccine.
Saya ingin dapatkan maklumat lanjut atau bukti saintifik tentang vaksin ini.

☐ I cannot afford to buy the vaccine. / *Saya tidak mampu untuk membeli vaksin ini*.

☐ I would rather practice preventive measurements to prevent dengue infection.
Saya lebih sanggup mengamalkan langkah-langkah pencegahan daripada dijangkiti penyakit denggi.

☐ Others, please specify: / *Lain-lain, sila nyatakan:*

____

Questions 15-21 for those who has children/ *Soalan 15-21 untuk mereka yang mempunyai anak.*

If only dengue vaccine B is available,
Jika vaksin denggi B sahaja yang ada,

15. Would you purchase dengue vaccine B for your children?
Adakah anda sanggup membeli vaksin B untuk anak-anak anda?

☐Yes/*Ya*
If yes, please proceed to No.16
Jika ya, sila teruskan ke No.16

☐No/*Tidak*
If no, please proceed to No.21
Jika tidak, sila teruskan ke No.21

16. Would you be willing to pay RM160 per dose for dengue vaccine B?
Adakah anda sanggup membayar RM160 setiap dos untuk vaksin denggi B?

☐Yes/*Ya*
If yes, please proceed to No.17
Jika ya, sila teruskan ke No.17

☐No/*Tidak*
If no, please proceed to No.19
Jika tidak, sila teruskan ke No.19

17. Would you be willing to pay RM240 per dose for dengue vaccine B?
Adakah anda sanggup membayar RM240 setiap dos untuk vaksin denggi B?

☐Yes/*Ya*
If yes, please proceed to No.18
Jika ya, sila teruskan ke No.18

☐No/*Tidak*

18. Would you be willing to pay RM320 per dose for dengue vaccine B?
Adakah anda sanggup membayar RM320 setiap dos untuk vaksin denggi B?

☐Yes/*Ya*
If yes, please proceed to No.20
Jika ya, sila teruskan ke No.20

☐No/*Tidak*

19. Would you be willing to pay RM 80 per dose for dengue vaccine B?
Adakah anda sanggup membayar RM 80 setiap dos untuk vaksin denggi B?

☐Yes/*Ya*

☐No/*Tidak*
If no, please proceed to No.20
Jika tidak, sila teruskan ke No.20

20. What is the maximum amount you are willing to pay for the vaccine per dose?
Apakah harga maksimum yang anda sanggup membayar untuk setiap dos vaksin denggi tersebut?

____

21. Please state the reason why you refuse to buy the vaccine.
Sila nyatakan sebab anda enggan membeli vaksin denggi tersebut.

☐ I would like to have free vaccination from government or claim from insurance company.
Saya ingin dapatkan vaksin percuma daripada kerajaan atau tuntut daripada syarikat insurans.

☐ I would like to have more information or scientific evidence on the vaccine.
Saya ingin dapatkan maklumat lanjut atau bukti saintifik tentang vaksin ini.

☐ I cannot afford to buy the vaccine. / *Saya tidak mampu untuk membeli vaksin ini*.

☐ I would rather practice preventive measurements to prevent dengue infection.
Saya lebih sanggup mengamalkan langkah-langkah pencegahan daripada dijangkiti penyakit denggi.

☐ Others, please specify: / *Lain-lain, sila nyatakan:*
____

How would you rate the difficulty of this questionnaire?
Berapakah tahap kesukaran soal selidik ini?

Very hard *Sangat susah*	Hard *Susah*	Neutral *Neutral*	Easy *Senang*	Very easy *Sangat senang*
1	2	3	4	5

---THE END/ *TAMAT*---
